# Development of Poly(lactic acid)-Based Biocomposites with Silver Nanoparticles and Investigation of Their Characteristics

**DOI:** 10.3390/polym16192758

**Published:** 2024-09-29

**Authors:** Kristine V. Aleksanyan, Regina S. Smykovskaya, Nadezhda A. Samoilova, Viktor A. Novikov, Aleksander M. Shakhov, Arseny V. Aybush, Olga P. Kuznetsova, Sergey M. Lomakin, Yana V. Ryzhmanova

**Affiliations:** 1 Higher Engineering School “New Materials and Technologies”, Plekhanov Russian University of Economics, 36 Stremyanny Ln, Moscow 117997, Russia; 2 Semenov Federal Research Center for Chemical Physics, Russian Academy of Sciences, 4 Kosygina St., Moscow 119991, Russia; 3 Nesmeyanov Institute of Organoelement Compounds, Russian Academy of Sciences, 28 Bld 1 Vavilova St., Moscow 119334, Russia; 4 Emanuel Institute of Biochemical Physics, Russian Academy of Sciences, 4 Kosygina St., Moscow 119991, Russia; 5 Skryabin Institute of Biochemistry and Physiology of Microorganisms, Federal Research Center “Pushchino Scientific Center for Biological Research of the Russian Academy of Sciences”, pr. Nauki 5, Pushchino 142292, Russia

**Keywords:** poly(lactic acid) (PLA), silver nanoparticles (AgNPs), thermal properties, structure, antibacterial activity

## Abstract

Nowadays, the demand for food packaging that maintains the safety and quality of products has become one of the leading challenges. It can be solved by developing functional materials based on biodegradable polymers, such as poly(lactic acid) (PLA). In order to develop PLA-based functional materials with antibacterial activity, silver nanoparticles (AgNPs) were introduced. In the present study, AgNPs stabilized by a copolymer of ethylene and maleic acid were used. Under the joint action of shear deformations and high temperature, the biocomposites of PLA with poly(ethylene glycol) and AgNPs were produced. Their mechanical and thermal characteristics, water absorption, and structure were investigated using modern methods (DSC, FTIR, Raman spectroscopy, SEM). The effect of AgNP concentration on the characteristics of PLA-based biocomposites was detected. Based on the results of antibacterial activity tests (against Gram-positive and Gram-negative bacteria, along with yeast) it is assumed that these systems have potential as materials for extending the storage of food products. At the same time, PLA–PEG biocomposites with AgNPs possess biodegradability.

## 1. Introduction

For more than two decades now, there has been a growing interest in polymers obtained from natural monomers due to their unique set of characteristics. In particular, polyesters of hydroxycarboxylic acids are widely investigated in terms of the formation of systems based on their application in different fields, such as packaging, medicine, agriculture, the textile industry, etc., for example, [1,2,3]. Among polymers of this class, poly(lactic acid) or polylactide (PLA) has proven itself as a polymer with properties close to synthetic polymers, despite its relatively high cost and fragility, but at the same time possessing biodegradability, for example, [4,5,6]. At present, PLA compositions with various polymers, such as natural ones, namely, polysaccharides [7,8,9,10], as well as synthetic [11,12,13,14], are often studied. However, of particular interest is research aimed at creating functional compositions based on PLA. It should be noted that only in recent years the studies on developing composites based on PLA with metal/metal oxide nanoparticles that have antimicrobial properties have become relevant since there is a need not only to create biodegradable materials but also to extend the shelf life of food products. In particular, there are investigations devoted to the preparation of materials based on PLA with uniformly dispersed ZnO nanoparticles by solvent volatilization [15]. It was shown that the introduction of nanoparticles led to an increase in elastic modulus and elongation at the break while, at the same time, a decrease in tensile strength. It is noted that this material inhibits microbial growth on products; that is, the effectiveness of its use as packaging has been proven [15]. It was shown in [16] that the addition of ZnO nanoparticles to PLA allows one to obtain materials that can potentially be used in food packaging due to their improved thermomechanical, UV protective, and antibacterial properties. The use of, for example, electrospinning also makes it possible to obtain such materials with improved antimicrobial and mechanical properties [17]. Vasile et al. [18] showed the possibility of obtaining new compositions of PLA with ZnO, Cu, and Ag nanoparticles by the direct synthesis of nanoparticles during mixing in the melt. The research on developing biodegradable materials based on polylactide with graphene oxide and silver nanoparticles should be highlighted [19,20]: the first component is added to improve mechanical characteristics, and the second to impart antimicrobial properties. Liu and Chan et al. [21] obtained from the melt a mixture of PLA with hydroxyapatite nanorods and silver nanoparticles; it was shown that a balance was found between antimicrobial activity and cytocompatibility; a biomineralization test showed the possibility of using such systems in bone engineering. Separately, a whole cycle of studies of compositions based on polylactide with nanocellulose and silver nanoparticles should be singled out [22,23,24,25,26]. However, the literature contains a limited number of studies devoted specifically to systems based on PLA with silver nanoparticles. Thus, in [27], the possibility of obtaining a sensor for detecting hydrogen peroxide based on polylactide coated with silver nanoparticles was shown, obtained by the reduction of silver from AgNO_3_ in dichloromethane by adding Piper nigrum leaves containing alkaloids. These systems can be used to determine even very low concentrations of hydrogen peroxide and can be used in biological and environmental analysis. In [28], triacetate glycerol was used as a plasticizer for polylactide, and a synergistic effect was revealed between silver nanoparticles and a plasticizer during the crystallization of PLA. It is shown that these mixtures have good mechanical, thermal, and optical properties. In this study, silver nanoparticles stabilized by a copolymer of ethylene and maleic acid, obtained by the technique in [29], were used. Biocomposites based on PLA plasticized with polyethylene glycol (PEG) were obtained under conditions of high temperatures and shear deformations in the absence of organic solvents. The aim of this study was to develop a biocomposite based on PLA possessing antibacterial properties, along with biodegradability, in order to create systems used as packaging materials, films, containers for storing and transporting food products, for use in agriculture, etc.

## 2. Materials and Methods

Poly(ethylene-alt-maleic anhydride) (Monsanto, Creve Coeur, MO, USA) with an average molecular weight M = 25,000 was used. The copolymer, before use, was hydrolyzed to the corresponding copolymers of maleic acid (EM) by dissolving in deionized water, followed by lyophilization (−55 °C, 0.05 mbar). The reagents NaBH_4_ (Sigma-Aldrich, Darmstadt, Germany), AgNO_3_, and NaOH (all of the analytical grade, “Reahim”, Moscow, Russia) were used without purification.

In experiments, PLA 4043D (NatureWorks, USA) with *T_m_* = 145–160 °C, density 1.24 g/cm^3^, and MFI of 6.0 g/10 min (210 °C under 2.16 kg loads), along with PEG (Sigma, Germany) of different molecular weights (1000, 4000), were used.

### 2.1. Synthesis of Silver Nanoparticles

The silver hydrosol EM/Ag0 was prepared by borohydride reduction of silver salt in the presence of EM, similar to [29]. Briefly, AgNO_3_, NaBH_4_, and polymer-stabilizer (EM) were dissolved in deionized water separately. Aqueous solutions of EM were prepared by weighing the solid copolymer, dissolving it in water at room temperature, and adding a 1M solution of NaOH for pH = 6 adjustment. The pH values were defined using a Fisher Scientific 300,403.1 pH meter (USA). Then, these solutions were vigorously stirred during and after (for 0.5 h) the addition of a 2-fold molar excess of aqueous NaBH_4_ at room temperature. Here, the molar concentration of EM refers to the monomeric maleic acid units. The reaction mixture was allowed to stand (24 h, 200 °C). Dried sample EM/Ag0 was obtained after ultrafiltration (cutoff 3.5 kDa) and freeze-drying (−55 °C, 0.05 mbar).

### 2.2. Biocomposite Production

Initial components were mixed under the action of shear deformations and high temperature in a twin-screw microextruder HAAKE Rheomex Minilab II (Thermo Scientific, Waltham, MA, USA). First, PLA was melted, then PEG or PEG with silver nanoparticles (AgNPs) was introduced into the PLA melt; the components were mixed at 170 °C for 10 min. The optimal rate of mixing was 50 rpm. After a lapse of time, the mixtures were extruded through a flat slit.

### 2.3. Pressing and Mechanical Tests

In order to test the biocomposite properties, the films with a width of ~0.3 mm were pressed on a Carver CH 4386.4010 (Wabash, IN, USA) laboratory press at 170 °C and 10 MPa for 10 min, followed by cooling under pressure at a rate of ~15 °C/min.

Mechanical tests of films were performed on an Instron-3365 (High Wycombe, UK) universal testing machine in a stretching mode at a rate of the upper traverse movement of 1 mm/min and at room temperature. Elastic modulus *E*, ultimate tensile stress *σ_b_*, and relative elongation at break *ε_b_* were calculated from the stress (*σ*)–strain (*ε*) diagrams. The results for 6–7 samples were averaged. The error of *E* and *σ_b_* did not exceed 10%, and the error of *ε_b_* was 5%.

### 2.4. Water Absorption

The water absorption test is based on an ASTM D570 standard [ASTM. Available online: https://www.astm.org/standards/d570 (accessed on 25 August 2024)]. In our case, the film samples of width ~0.3 mm were placed in distilled water and kept in a thermostat at room temperature for some period of time. The samples were weighed at certain time intervals.

### 2.5. SEM Analysis

The samples were cut as 5 × 5 mm sheets and placed onto electro-conductive carbon tape adhered to the SEM sample holder. The samples for cross-section imaging were obtained by liquid nitrogen cooling, followed by the brittle fracture of the samples. A gold layer of 10 nm thickness was deposited on the sample surface inside spatter coater Q 150R ES (Quorum, Barcelona, Spain), followed by sample loading inside a chamber of a Prisma E (Thermo Fisher Scientific, Waltham, MA, USA) scanning electron microscope. SEM imaging was performed in secondary electron mode at low voltage settings (0.5–1.5 kV) to demonstrate the surface features of each sample.

### 2.6. Spectral Analysis

The samples were characterized in terms of absorption and vibration spectroscopy. Absorption spectra of AgNPs were measured by the spectrophotometer Shimadzu UV-3600 (Shimadzu, Tokyo, Japan). Raman spectra were collected by the microscopy module of an FRCCP RAS laser setup: the laser irradiation was focused by a Mitutoyo (Mitutoyo, Kawasaki city, Japan) objective with NA = 0.42, and the scattered light was filtered (LP03-458RU-25, Semrock, Rochester, NY, USA) and registered by CCD (Andor Newton 920, Oxford Instruments, Oxford, UK). The excitation wavelength was chosen to be at 457 nm (CNI, Changchun, China), close to the absorption band of AgNP. FTIR measurements were made using a LUMOS-II (Bruker, Berlin, Germany) spectrometer in ATR mode. Both Raman and FTIR spectra were collected from multiple parts to deal with sample heterogeneity on the micro scale.

### 2.7. DSC Analysis

The thermophysical characteristics of PLA-based samples were studied using the DSC method on a DSC-204 F1 calorimeter (NETZSCH-Gerätebau GmbH, Selb, Bavaria, Germany) at a heating rate of 10 K/min in an inert atmosphere of Ar in the temperature range of 20–200 °C.

Typically, in DSC studies of polymers, it is customary to use a repeating heating–cooling mode to remove the “prehistory” of their formation. In this work, DSC studies were carried out in a single-step mode without reheating since we intended to characterize the primary morphology of the PLA samples, not “erase their thermal memory” or “thermodynamically balance” their original structure [30].

The degree of crystallinity of PLA samples, *χ*%, was calculated by the equation

$$\chi =\frac{\Delta H_{m}-\Delta H_{cc}}{\Delta H_{m}^{100}(1-\alpha )}\times 100\%$$

where $\Delta H_{m}$—enthalpy of melting, $\Delta H_{cc}$—enthalpy of crystallization (enthalpy of “cold” crystallization not observed in our case), *α*—mass fraction of PEG, and $\Delta H_{m}^{100}$—the theoretical value of the 100% crystalline poly(L-lactide) melting enthalpy (93.6 J/g) [31].

By analogy with PLA, the degree of crystallinity of PLA samples, *χ*%, was calculated by the equation

$$\chi =\frac{\Delta H_{m}}{\Delta H_{m}^{100}}\times 100\%$$

where $\Delta H_{m}^{100}$ is the melting enthalpy of 100% crystalline PEG (213.0 J/g) [32].

### 2.8. Test of Antibacterial Activity

The different types of microorganisms were used to carry out the test of antibacterial activity (Table 1). *B. subtilis*, *E. coli*, and *M. luteus* were grown on the following medium: aminopeptide 60 mL; trypton 5.0 g; yeast extract 1.0 g; soybean extract 30 mL; bacto-agar 15.0 g; final pH 7.2. *G. auringiensis* was grown on malt extract medium containing the following (g/L): malt extract 12.75; dextrin 2.75; glycerol 2.35; gelatin peptone 0.78; bacto-agar 15; final pH 5.4. *C. sporogenes* was cultivated anaerobically under 100% nitrogen atmosphere on a medium containing the following (g/L): trypticase 5.0; peptone 5.0; yeast extract 10.0; salt solution 40.0 mL; Na-resazurin (0.1% *w*/*v*) 0.5 mL; L-cysteine-HCl 0.5; Na_2_CO_3_ 2.5; D-glucose 5.0; bacto-agar 15.0. The salt solution contained the following (g/L): CaCl_2_ × 2H_2_O, 0.25; MgSO_4_ × 7H_2_O 0.5; K_2_HPO_4_ 1.0; KH_2_PO_4_ 1.0; NaHCO_3_ 10.0; and NaCl 2.0.

Microorganisms were applied to the surface of the solid medium in the amount of 10^10^ CFU. Then, film samples with a size of approximately 0.5 × 0.5 cm, pre-treated in 70% ethanol, and washed with sterile distilled water were placed on the surface of the agar. Cultivation was carried out at 30 °C for 120 h in two replicates. *B. subtilis*, *E. coli*, *M. luteus*, and *G. auringiensis* were cultivated aerobically, but *C. sporogenes* was grown in an anaerobic jar under a 100% nitrogen atmosphere. The inhibitory capacity of the film samples was assessed by the presence of bacterial and yeast growth around film samples and under the one. The photos were taken using a standard photo camera.

### 2.9. Biodegradability

The biodegradability of biocomposites was studied by imitating the environmental conditions according to a technique based on an ASTM D5988-12 standard. For this aim, the samples under investigation were placed into a container with wet soil (pH 6–7) designated for plant growth (OOO “Russkaya Torfyanaya Kompaniya”). The soil composition was as follows: high-moor peat, agroperlite, biohumus, dolomite dust, complex liquid fertilizer, and natural zeolite. Containers were incubated at room temperature for several months. The biodegradation rate was estimated by the mass loss of samples determined at certain time periods.

## 3. Results and Discussion

### 3.1. PLA-Based Biocomposites

**Figure 1:**
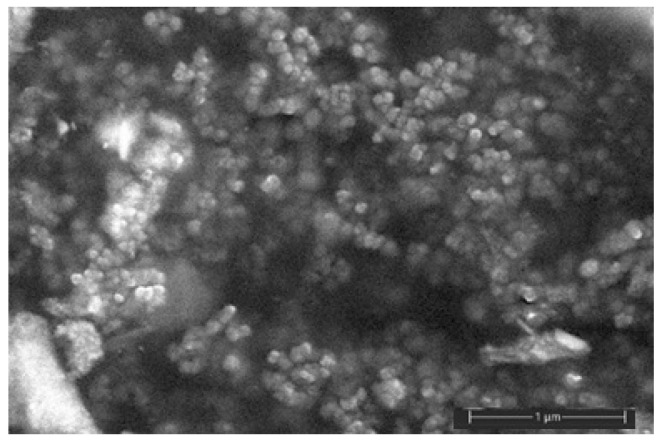
SEM micrograph of AgNP powder.

In order to synthesize AgNPs, we used a simple method for the production of stable silver hydrosols based on the reduction of metal-precursor ions in the presence of maleic acid (anhydride) copolymer with NaBH_4_ in aqueous solutions at room temperature. The main advantages of maleic acid (anhydride) copolymers and, in particular, copolymer poly(ethylene-alt-maleic acid (anhydride)) are a regular structure of polymeric chain (strict alternation of comonomer units), commercial availability, and solubility in water. This copolymer has been successfully used to stabilize the nanoparticles of a number of noble metals and metal oxides [33,34]. According to the synthesis technique, a golden-brown powder of stabilized AgNPs was obtained. Figure 1 illustrates the SEM micrograph of dried AgNP powder: the shape of the nanoparticles is close to spherical, and the size is about 40–60 nm.

PLA-based biocomposites with and without AgNPs were obtained in a twin-screw microextruder under the joint action of shear deformation and high temperature. First, PLA granules were loaded into an extruder to obtain the polymer melt, and then PEG or PEG mixed with AgNPs was introduced into the PLA melt. The PLA content was varied from 80 to 90 wt%, PEG from 10 to 20 wt%, and AgNPs from 0.01 to 0.5 wt% by the PLA–PEG biocomposite weight. In order to explore the properties of the final biocomposites, the films were pressed.

### 3.2. Mechanical Characteristics, Water Absorption, and Structural Features

In order to affect the mechanical properties of PLA, the plasticizer, PEG, of different molecular weights was introduced. The characteristics of the binary compositions were lower than those for pure PLA, and PEG sweats out in time [6]. The mechanical characteristics of the PLA–PEG biocomposites with AgNPs are presented in Table 2. As was expected, these data are characteristic of glassy polymers with low elongation at break. It can be seen from the table that the rise in AgNP content from 0.01 to 0.5 wt% led to a substantial rise in elastic modulus and tensile strength, as well as an insignificant increase in elongation at break. At maximal AgNP concentration, the characteristics are comparable to those of neat PLA, for example, [35].

**Figure 2:**
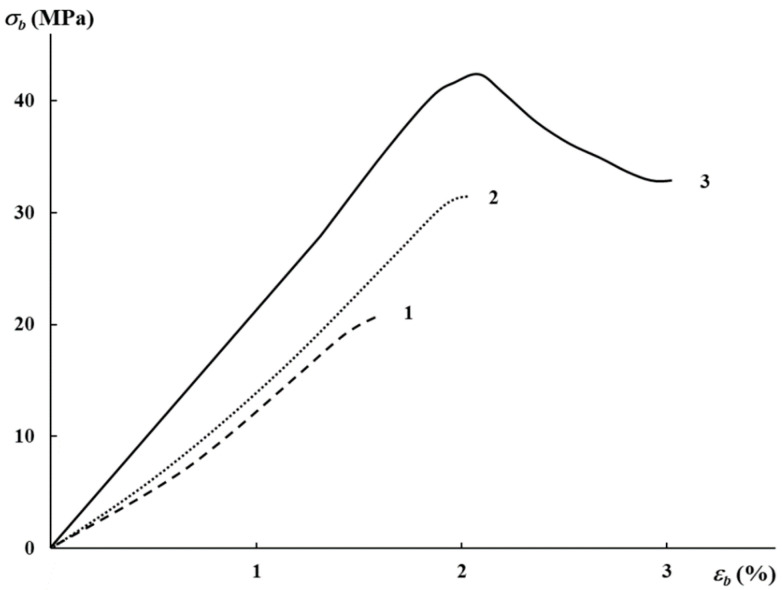
Stress–strain diagrams of PLA–PEG_4000_ biocomposites with AgNPs (wt%): 0.01 (1), 0.03 (2), 0.5 (3).

Figure 2 illustrates the stress–strain diagrams for the PLA–PEG_4000_ + AgNPs biocomposites at different content of AgNPs. As is seen, only at 0.5 wt% content of AgNPs does the neck begin to form at 2% elongation on the deformation curve, which is characteristic of initial PLA. The further deformation of the sample led to a change in the section of initial and narrowed zones; the neck is spread over the biocomposite sample. Thus, the introduction of AgNPs leads to a rise in mechanical characteristics.

**Figure 3:**
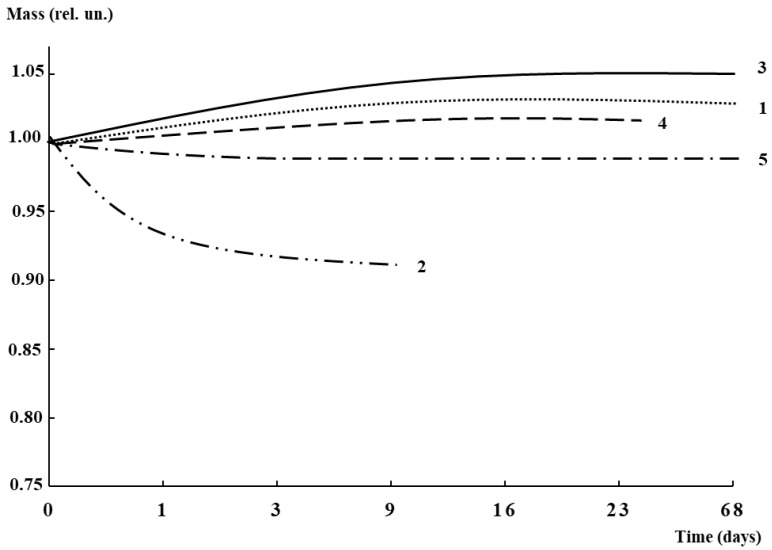
Water absorption curves for PLA-based biocomposites (wt%): PLA–PEG_1000_ (90:10) (1), PLA–PEG_1000_ (80:20) (2), PLA–PEG_4000_ + AgNPs (90:10 + 0.01) (3), PLA–PEG_1000_ + AgNPs (90:10 + 0.1) (4), PLA–PEG_4000_ + AgNPs (90:10 + 0.2) (5).

Since water absorption is an indirect factor of biodegradability and due to the presence of water-soluble plasticizer in the biocomposites, this parameter was evaluated according to an international standard. Figure 3 illustrates the characteristic curves of water absorption for PLA-based biocomposites, both with AgNPs and without them. As was expected, the PLA–PEG_1000_ (80:20 wt%) films with maximal content of plasticizer began falling apart in the first 24 h, then it decomposed (there was no possibility to collect the pieces) completely in nine days. This is connected with the fact that the higher the PEG content, the more loose the biocomposite structure. Therefore, the optimal PEG content was chosen at 10 wt%. The maximal water absorption for the biocomposites, both with AgNPs and without them, was achieved after 9–15 days of the test, then the factor achieved a plateau, and there was no further rise in this factor. The maximum water absorption was observed for the system with the minimal AgNP content.

**Figure 4:**
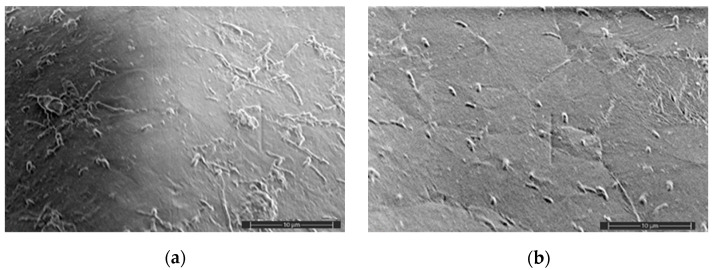

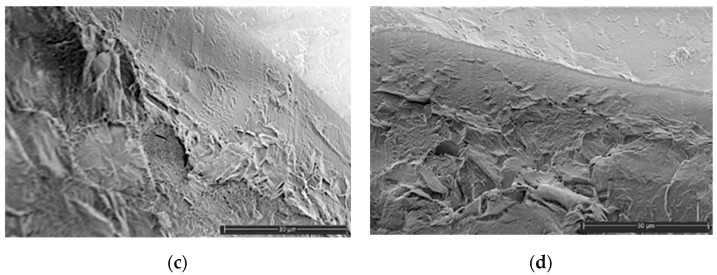
SEM micrographs of the sample surface (**a**,**b**) and cross-sections (**c**,**d**) after tests of water absorption at different concentrations of AgNPs (×5000): PLA–PEG_1000_ + AgNPs (90:10 + 0.1 wt%) (**a**,**c**); PLA–PEG_4000_ + AgNPs (90:10 + 0.2 wt%) (**b**,**d**). Images are presented at different magnification: 5000 (**a**,**b**); 2500 (**c**,**d**).

The surface morphology of the samples after water absorption was also investigated with the use of the SEM method since the plasticizer was washing out (Figure 4). The samples were dried before the test, and then the optimal conditions of imaging were selected. Independently on the AgNP concentration, the micrographs are characterized by similar surface morphology. However, it should be noted that only the system with 0.2 wt% of AgNPs had surface cracks with an insignificant depth and a 5–15 μm length (Figure 4b).

**Figure 5:**
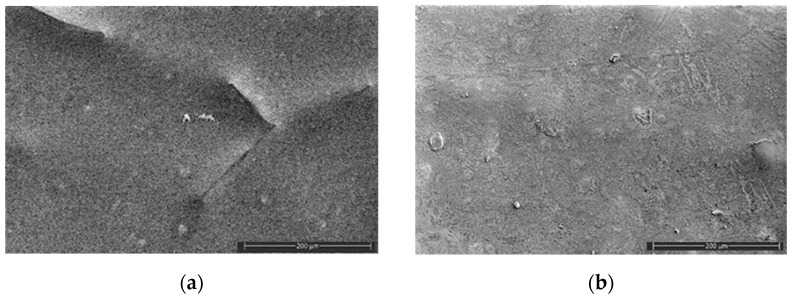
SEM micrographs of the sample surface at different AgNP concentrations (×350): PLA–PEG_4000_ + AgNPs (90:10 + 0.2 wt%) (**a**); PLA–PEG_4000_ + AgNPs (90:10 + 0.5 wt%) (**b**).

The analysis of the cross-sections of the samples after water absorption did not illustrate any substantial deterioration of the films; however, there was no sign of nanoparticle agglomeration (Figure 4c,d). However, after exposure to water, the films are defective, in contrast with the initial films, the surface SEM images of which are presented in Figure 5.

The comparison of the surface micrographs of the initial films at different concentrations of the AgNPs showed that the nanoparticles are visualized both on the surface and in the bulk of the biocomposite. It is evident that the nanoparticles are uniformly distributed in the PLA matrix (Figure 5). Thus, the mixing of the biocomposites under the action of shear deformations did not result in substantial agglomeration of AgNPs.

### 3.3. Spectral Analysis

**Figure 6:**
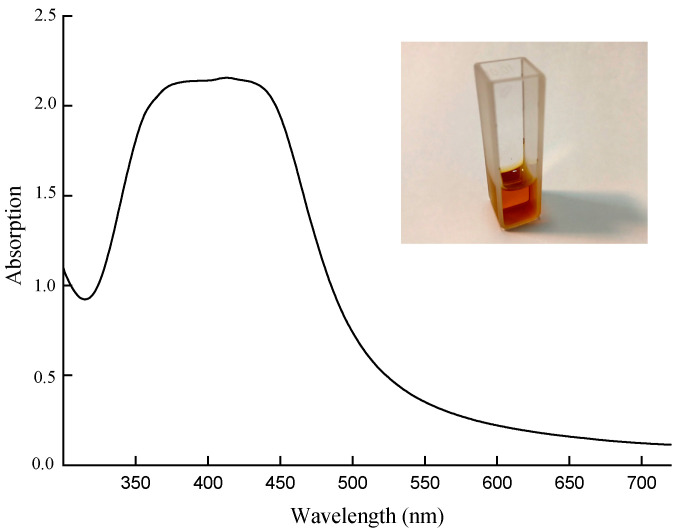
Absorption of AgNPs. Photo: colloid.

The powder of AgNPs was dissolved in deionized water, and then the solution was subjected to intense ultrasound for 30 min to reduce the fraction of aggregates, followed by centrifugation at 14,000 rpm for 30 min. The absorption spectrum of the resulting solution in the range of 300–700 nm is shown in Figure 6. Spherical AgNPs (which are known to have a peak position at 420 nm in water) dominate the colloid, though the plateau of the absorption band indicates a possible residual contribution from aggregates.

**Figure 7:**
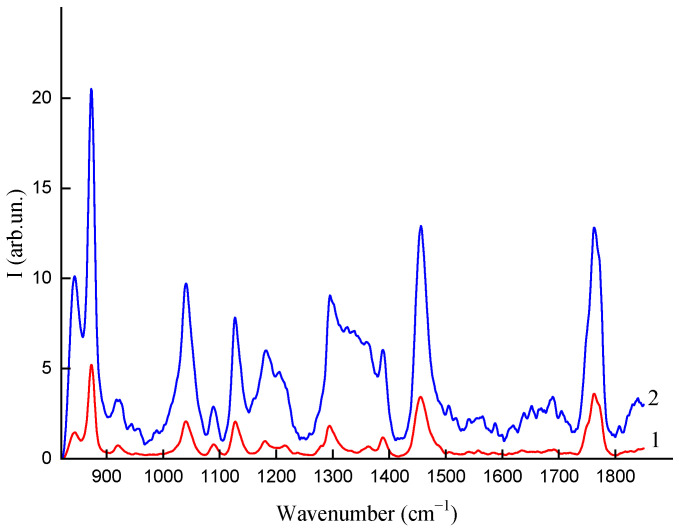
Raman spectra: PLA–PEG_4000_ + AgNPs (90:10 + 0.02 wt%) (1); PLA–PEG_4000_ + AgNPs (90:10 + 0.2 wt%) (2).

**Figure 8:**
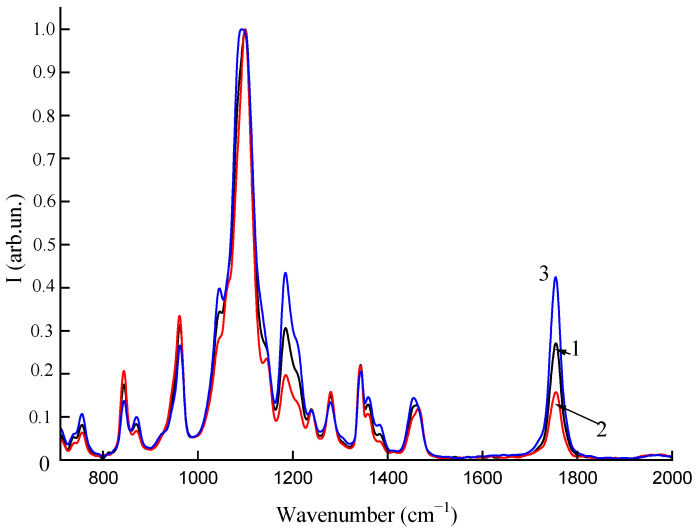
FTIR spectra: PLA–PEG_1000_ + AgNPs (90:10 + 0.1 wt%) (1); PLA–PEG_4000_ + AgNPs (90:10 + 0.02 wt%) (2); PLA–PEG_4000_ + AgNPs (90:10 + 0.2 wt%) (3).

The average Raman spectra of the samples with two different fractions of AgNPs are shown in Figure 7. Data averaging was performed over a large sample area, and several hundred spectra were collected. As can be seen from Figure 7, the intensity increase of the Raman bands follows the fraction of AgNPs, which can be explained by the SERS effect of AgNPs showing the polymer composition at a nano scale. The average enhancement factor of a single AgNP is reversely proportional to the fraction of AgNPs, and it can be assessed as ~1000. In contrast, AgNPs cannot contribute to signal enhancement in FTIR measurements (Figure 8). Both vibrational bands of Raman and FTIR spectroscopy give spectra that are similar in structure in terms of the main vibrational bands. The FTIR spectra normalized by the main band of 1100 cm^−1^ are shown for different fractions (0.02, 0.1, 0.2%) of AgNPs in Figure 8. It can be seen from the figure that the fraction increase affects the C=O band (~1780 cm^−1^) of carboxylic acids several times. Thus, both Raman and FTIR show sensitivity to the effect of AgNP concentration in polymer films.

### 3.4. Differential Scanning Calorimetry

**Figure 9:**
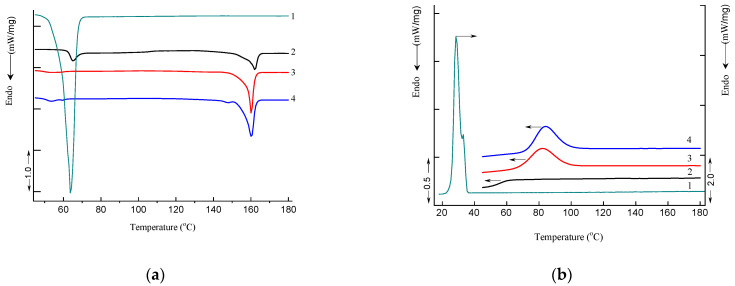
DSC heat flow curves of heating (**a**) and cooling (**b**) for PEG_4000_ (1), PLA (2), PLA–PEG_4000_ (90:10 wt%) (3), PLA–PEG_4000_ + AgNPs (90:10 + 0.2 wt%) (4).

Figure 9 shows DSC thermograms of PLA, PEG, PLA–PEG_4000_ (90:10 wt%), and PLA–PEG_4000_ + AgNPs (90:10 + 0.2 wt%) samples obtained in heating and cooling modes. The DSC parameters include the following: glass transition temperature *T_g_*, peak temperature of enthalpy relaxation *T_r_*, crystallization temperature *T_cr_* (cooling), melting temperature *T_m_*, characteristic enthalpies of crystallization Δ*H_cr_* (cooling, based on PEG mass fraction of 0.1) and melting Δ*H_m_*, and degree of crystallization (*χ*); these are summarized in Table 3.

Small “relaxation” effects in PLA and PLA–PEG_4000_ + AgNPs (90:10 + 0.2 wt%) samples illustrated by endothermic heat capacity peaks (*T_r_*) can be seen at temperatures above the *T_g_* (Figure 9a). These peaks characterize the phenomenon of enthalpy relaxation, which is associated with the mobility and the recovery of PLA chains to their thermodynamic equilibrium status at a temperature above *T_g_*.

Analysis of DSC data showed that the degree of crystallinity of PLA increases more than twice in the presence of a PEG plasticizer (Table 3). The addition of AgNPs in the composition with PLA–PEG_4000_ results in the formation of low-temperature disordered (hexagonal) crystalline forms of PLA with a melting temperature of 148.1 °C due to the nucleation effect of AgNPs (Figure 9a). The DSC results clearly demonstrate that AgNPs and PEG can act as high-efficiency nucleating agents and plasticizers. This fact is especially clearly manifested in the cooling curves of the PLA–PEG_4000_ (90:10 wt%) and PLA–PEG_4000_ + AgNPs (90:10 + 0.2 wt%) samples, in contrast to the pristine PLA sample (Figure 9b). These DSC curves clearly show the exothermic process of PLA crystallization upon cooling (*T_cr_* = 82.6 and 83.9 °C, *ΔH_cr_* = 33.2 and 33.1 J/g for PLA–PEG_4000_ (90:10 wt%) and PLA–PEG_4000_ + AgNPs (90:10 + 0.2 wt%) samples, respectively).

### 3.5. Antibacterial Activity and Biodegradability

**Figure 10:**
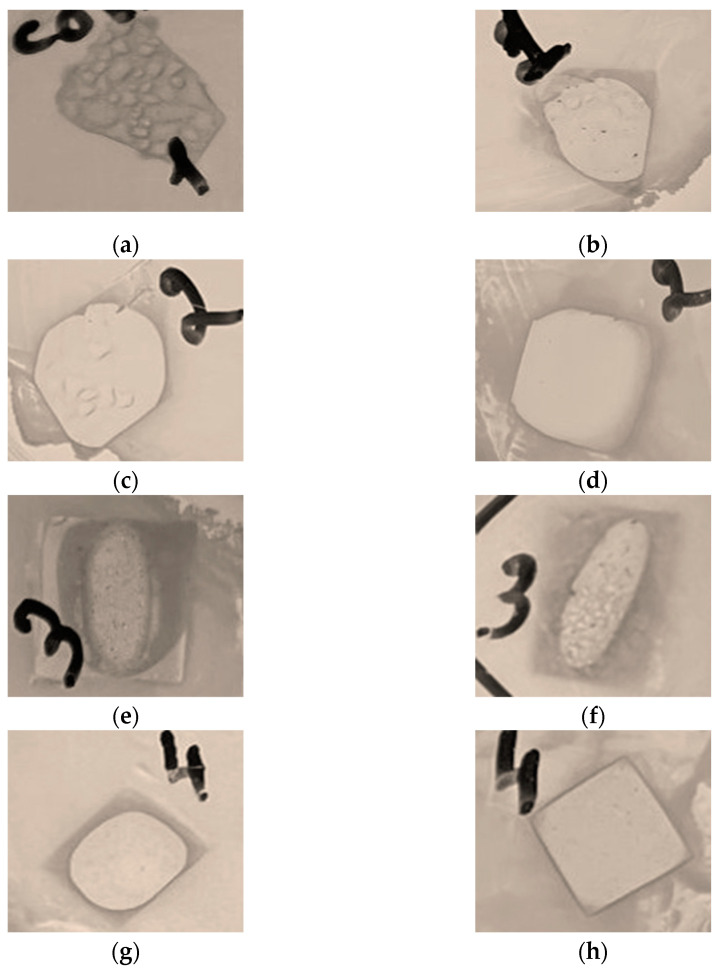
Photos of PLA–PEG + AgNPs films in media with different bacteria. PEG_1000_ (**a**–**d**); PEG_4000_ (**e**–**h**). AgNP concentration (wt%): 0.1 (**a**,**b**,**e**,**f**); 0.5 (**c**,**d**,**g**,**h**). *E. coli* (**a**,**g**), *B. subtilis* (**b**,**c**,**f**), *M. luteus* (**d**,**h**), *C. sporogenes* (**e**).

It should be noted that the individual components, namely PLA and PEG, do not inhibit microorganism growth, which is repeatedly demonstrated in previous investigations, for example, [36,37,38]. In terms of biocomposite films, the zones of clearing around them were not observed with any microorganisms, which probably means that the components of the films do not migrate into the environment. However, the zones of clearing were under the films, where the surface contact with the medium was greater (Figure 10). There was no growth of *G. auringiensis*, *M. luteus*, or *B. subtilis* under the film samples, which indicates a complete inhibitory effect for these microorganisms. In the case of *E. coli*, weak growth was observed only under the PLA–PEG_1000_ + AgNPs (90:10 + 0.1 wt%) film, probably due to the insufficient antimicrobial activity of the sample components. With other film samples, the growth of *E. coli* was absent. The weak growth of *C. sporogenes* was observed with all film samples (Table 4, Figure 10). This is probably due to the fact that *Clostridia* exhibit two mechanisms for tolerance of heavy metals, including reductive precipitation and the formation of heavy metal–protein complexes [39,40]. The results of the experiment are presented in Table 4.

**Figure 11:**
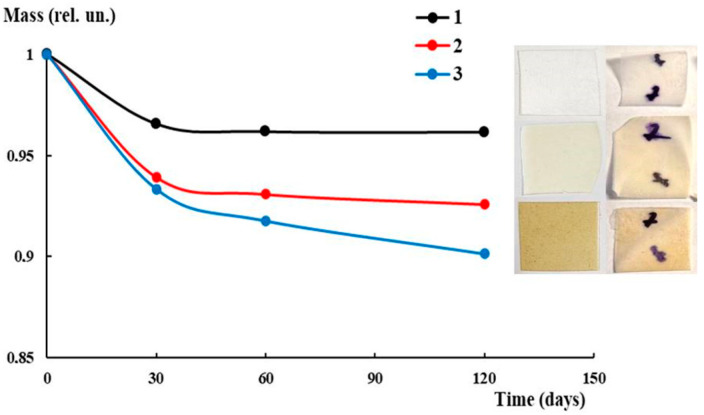
Mass loss curves of biocomposites after exposure in soil: PLA–PEG_4000_ (90:10 wt%) (1); PLA–PEG_4000_ + AgNPs (90:10 + 0.1 wt%) (2); PLA–PEG_4000_ + AgNPs (90:10 + 0.5 wt%) (3). Photos of films before (left column) and after (right column) test in the same order as curves.

In order to evaluate the possibility of biodegradation for the PLA-based biocomposites under investigation, the tests on biodegradability were carried out under conditions imitating nature (joint action of soil microorganisms, water, sunlight). Figure 11 illustrates the mass loss curves with the corresponding photos of the samples without and with AgNPs before and after the test. The most pronounced decrease in mass for all systems was observed in the first month, which is caused, to some extent, by the washing out of PEG. As is seen from the figure, in the case of plasticized PLA without AgNPs, the mass loss did not exceed 5%. The only change detected was the slight bending of the film after exposure to soil. In turn, the introduction of AgNPs led to some intensification of the mass loss. The maximal loss (about 10%) was detected for the PLA–PEG_4000_ + AgNPs (90:10 + 0.5 wt%) biocomposite after 4 months. The very beginning of the investigation of biocomposite biodegradability showed the principal potential for the possibility of degrading under environmental conditions.

## 4. Conclusions

In order to meet modern demands for developing functional materials based on polymers applied in food packaging and storage, PLA-based biocomposites with PEG and AgNPs were produced. Using an ethylene copolymer of maleic acid, stabilized AgNPs were obtained with a spherical shape and a size of 40–60 nm. Under the joint action of shear deformation and high temperature, the biocomposites of PLA with PEG and AgNPs at different component ratios were obtained in a twin-screw microextruder, and then the films were pressed. The effect of AgNPs on mechanical characteristics showed that the rise in AgNP content led to a rise in elastic modulus, tensile strength, and elongation at breakup compared to those for neat PLA. The presence of water-soluble PEG affected the water absorption ability (indirect characteristic of biodegradability): the higher the PEG content, the greater the index. The investigation of the biocomposite morphology using an SEM method allowed for assessing the uniform distribution of AgNPs in the biocomposite. Despite the low concentration of AgNPs in the PLA–PEG biocomposites, both Raman and FTIR showed sensitivity to the effect of AgNP concentration. According to the results of the DSC method, AgNPs and PEG can act as high-efficiency nucleating agents and plasticizers. This fact is especially clearly manifested in the cooling curves of the PLA–PEG_4000_ (90:10 wt%) and PLA–PEG_4000_ + AgNPs (90:10 + 0.2 wt%) biocomposites. During the investigation of antibacterial activity, the Gram-positive (*B. subtilis*, *M. luteus*, *C. sporogenes*) and Gram-negative (*E. coli*) bacteria, along with yeast (*G. auringiensis*), were used. It was found that these biocomposites inhibit microbial growth. At the same time, tests on biodegradability showed the principal possibility of subjecting them to biodegradation under natural conditions. However, this aspect requires further detailed investigation using more specific approaches (compost, UV-irradiation). Thus, the PLA–PEG + AgNPs biocomposites can be promising materials used not only for packaging and storage of food products but also in agriculture and medicine.

## Figures and Tables

**Table 1:** Microorganisms used in antibacterial activity test.

Microorganism	Type of Microorganism
*Bacillus subtilis* VKM B-501	Gram-positive obligate aerobic bacterium
*Escherichia coli* VKM B-3674	Gram-negative facultative anaerobic bacterium
*Micrococcus luteus* VKM Ac-2230	Gram-positive obligate aerobic bacterium
*Clostridium sporogenes* VKM B-2623	Gram-positive obligate anaerobic bacterium
*Groenewaldozyma auringiensis* VKM Y-2623	Facultative anaerobic yeast

**Table 2:** Mechanical characteristics of the PLA–PEG_4000_ + AgNPs biocomposites. The PLA to PEG_4000_ ratio was 90:10 wt%.

AgNP Content, wt%	*E*, MPa	*σ_b_*, MPa	*ε_b_*, %
0.01	2130	19.8	1.5
0.03	2370	31.7	2
0.5	3050	32.0	3

**Table 3:** DSC parameters of thermal transitions observed in PLA, PEG, PLA–PEG_4000_ (90:10 wt%), and PLA–PEG_4000_ + AgNPs (90:10 wt% + 0.2 wt%).

Sample		*T_g_*, °C	*T_r_*, °C	*T_m_*, °C PEG	*T_cr_*, °C PEG	*T_cr_*, °C PLA	*T_m_*, °C PLA	Δ*H_cr_*, J/g	Δ*H_m_*, J/g	χ, %
PLA	heating	63.7	65.1	-	-	-	162.0	-	−17.5 PLA	18.8
	cooling	57.0	-	-	-	-	-	-	-	-
PEG	heating	-	-	63.9	-	-	-	-	−212.4 PEG	99.7
	cooling	-	-	-	32.7/28.4	-	-	170.1 PEG	-	-
PLA–PEG_4000_ (90:10 wt%)	heating	50.8	-	-	-	-	160.2	-	−42.1 PLA	45.0
	cooling	-	-	-	-	82.6	-	33.2 PLA	-	-
PLA–PEG_4000_ + AgNPs (90:10 wt% + 0.2 wt%)	heating	51.9	-	59.8	-	-	160.3/ 148.0 *	-	−42.4 PLA	45.3
	cooling	-	-	-	-	83.9	-	33.1 PLA	-	-

* refer to the α′-limit disordered (hexagonal) crystalline forms of PLA.

**Table 4:** Growth of test cultures under the film samples.

Sample		*G. auringiensis*	*E. coli*	*M. luteus*	*B. subtilis*	*C. sporogenes*
PLA–PEG_1000_ + AgNPs	90:10 wt% + 0.1 wt%	no growth	weak growth	no growth	no growth	weak growth
	90:10 wt% + 0.5 wt%	no growth	no growth	no growth	no growth	weak growth
PLA–PEG_4000_ + AgNPs	90:10 wt% + 0.1 wt%	no growth	no growth	no growth	no growth	weak growth
	90:10 wt% + 0.5 wt%	no growth	no growth	no growth	no growth	weak growth

## Data Availability

Data is contained within the article.
